# The role of nucleic acid sensors in antifungal immunity

**DOI:** 10.3389/fimmu.2025.1725717

**Published:** 2025-12-11

**Authors:** Xinyu Liang, Hui Zhou, Litong Ouyang, Jiming Chen, Wenji He

**Affiliations:** 1 Key Laboratory of Innate Immunity and Chronic Inflammatory Diseases, Jiangxi Provincial Department of Education, Gannan Medical University, Ganzhou, China; 2 School of Basic Medicine, Gannan Medical University, Ganzhou, China

**Keywords:** nucleic acid sensors, fungal immunity, immune escape, ubiquitination regulation, type -1 interferons

## Abstract

Fungal infections pose a grave threat to individuals with compromised immune systems, and the accelerated proliferation of drug-resistant strains has led to a marked decline in the effectiveness of conventional antifungal medications in clinical settings. Achieving a more profound comprehension of the mechanisms underlying the host-pathogen interaction is imperative for the effective management of such infections. This review methodically elucidates the pivotal role of nucleic acid sensors as a pivotal subclass of pattern recognition receptors in antifungal immunity, their regulatory networks, and their competitive relationship with pathogen escape strategies. The present study focuses on cytoplasmic and endosomal nucleic acid sensors, delving into their critical roles in antifungal immunity and elucidating three aspects: recognition mechanisms, host regulatory mechanisms, and fungal escape. The results demonstrate that the functions of nucleic acid sensors exhibit significant pathogen specificity, reflecting their personalized and precise roles in antifungal immunity. Furthermore, within the regulatory mechanisms of nucleic acid sensors in the host, the processes of ubiquitin modification and autophagy pathway signaling balance are of significant importance. Concurrently, fungi have been observed to circumvent immune defenses through modifications to their cell walls and the secretion of immunosuppressive factors. This study reveals that the dynamic interplay between the Nucleic acid sensor network and fungal escape strategies holds clinical application potential, providing theoretical support and directional recommendations for clinical immune intervention strategies targeting ubiquitinylation nodes, cell death effector molecules, and other drug-resistant fungi.

## 1 Introduction

The severity of invasive fungal infections far exceeds previous recognition. Annually, these infections, including invasive forms and chronic pulmonary aspergillosis, affect over 6.5 million individuals and are responsible for approximately 3.8 million deaths. Critically, the vast majority of fatalities originate from undiagnosed and untreated cases. This reveals a substantial global shortfall in diagnostic capabilities and healthcare accessibility for fungal diseases, rendering it a severely underestimated public health crisis (1). There is an urgent need to deepen the understanding of host-fungal interaction mechanisms to develop novel strategies for prevention and treatment (2). In the battle against fungal incursion, the host’s intrinsic immune system functions as the primary line of defense, with its fundamental role being the identification of pathogen-associated molecular patterns (PAMPs) by pattern recognition receptors (PRRs). Nucleic acid sensors, a pivotal subclass of PRRs, are capable of discerning fungal nucleic acids, thereby instigating a series of downstream signaling cascades. These cascades orchestrate the release of inflammatory factors, the activation of cell death programs, and the initiation of adaptive immunity, culminating in the eradication of the pathogen (3).

The classification of nucleic acid sensors is predicated on their capacity to recognize fungal nucleic acid components. The two categories of sensors are DNA sensors and RNA sensors. These cells have been observed to activate specific signaling pathways through a process of differential recognition of fungal nucleic acid structures, including dsDNA, CpG motifs, and dsRNA (**Table 1**). It is noteworthy that these pathways exhibit a “double-edged sword” characteristic in antifungal immunity. Moderate activation can eliminate pathogens, but excessive activation may cause tissue damage and even be hijacked by fungi to promote infection (4).

**Table 1:** Summary of major nucleic acid sensors and their roles in antifungal immunity.

Sensor	Fungal ligand	Key effector molecules	Primary immune function	Key fungal pathogens
cGAS	dsDNA	Type I IFNs, CXCL10, TNF-α,IL-6,IL-1β	Mounting a robust type I interferon response	*Candida albicans (*5), *Aspergillus fumigatus (*6)
AIM2	dsDNA	Mature IL-1β, IL-18, Pyroptosis	Inflammasome activation and pyroptotic cell death	*Talaromyces marneffei (*7)*, Candida albicans (*8), *Aspergillus fumigatus (*9)
ZBP1	Z-RNA	RIPK3, RIPK1, Caspase-8, PANoptosis	Necroptosis, Apoptosis, Proinflammatory cytokines	*Candida albicans*, *Aspergillus fumigatus (*10)
MDA5	dsRNA	Type I IFNs, MAVS	Activation of downstream signaling pathways through the linker protein MAVS induces the production of interferon type I and inflammatory factors	*Candida albicans (*11)
TLR3	dsRNA	Type I IFNs, TNF-α, IL-6	Inducing anti-inflammatory and antiviral responses	*Candida albicans (*12), *Aspergillus fumigatus (*13)
TLR7/8	ssRNA	Type I IFNs, TNF-α, IL-6, IL-12	Promoting pro-inflammatory and Th1-polarizing responses	*C.albicans (*14), *A. fumigatus (*15), *Cryptococcus neoformans (*16)
TLR9	Unmethylated CpG DNA	Type I IFNs, TNF-α, IL-6, IL-12	Initiating pro-inflammatory cytokine production	*C. albicans*, *A. fumigatus (*17)

This review focuses on the central role of nucleic acid sensors in antifungal immunity. It systematically analyzes their recognition mechanisms, signaling networks, and the interplay with fungal evasion strategies. The aim of this analysis is to provide a theoretical basis for novel antifungal therapies targeting immune regulation.

## 2 Main types of nucleic acid sensors

### 2.1 DNA sensors

#### 2.1.1 cGAS-STING pathway

**Figure 1:**
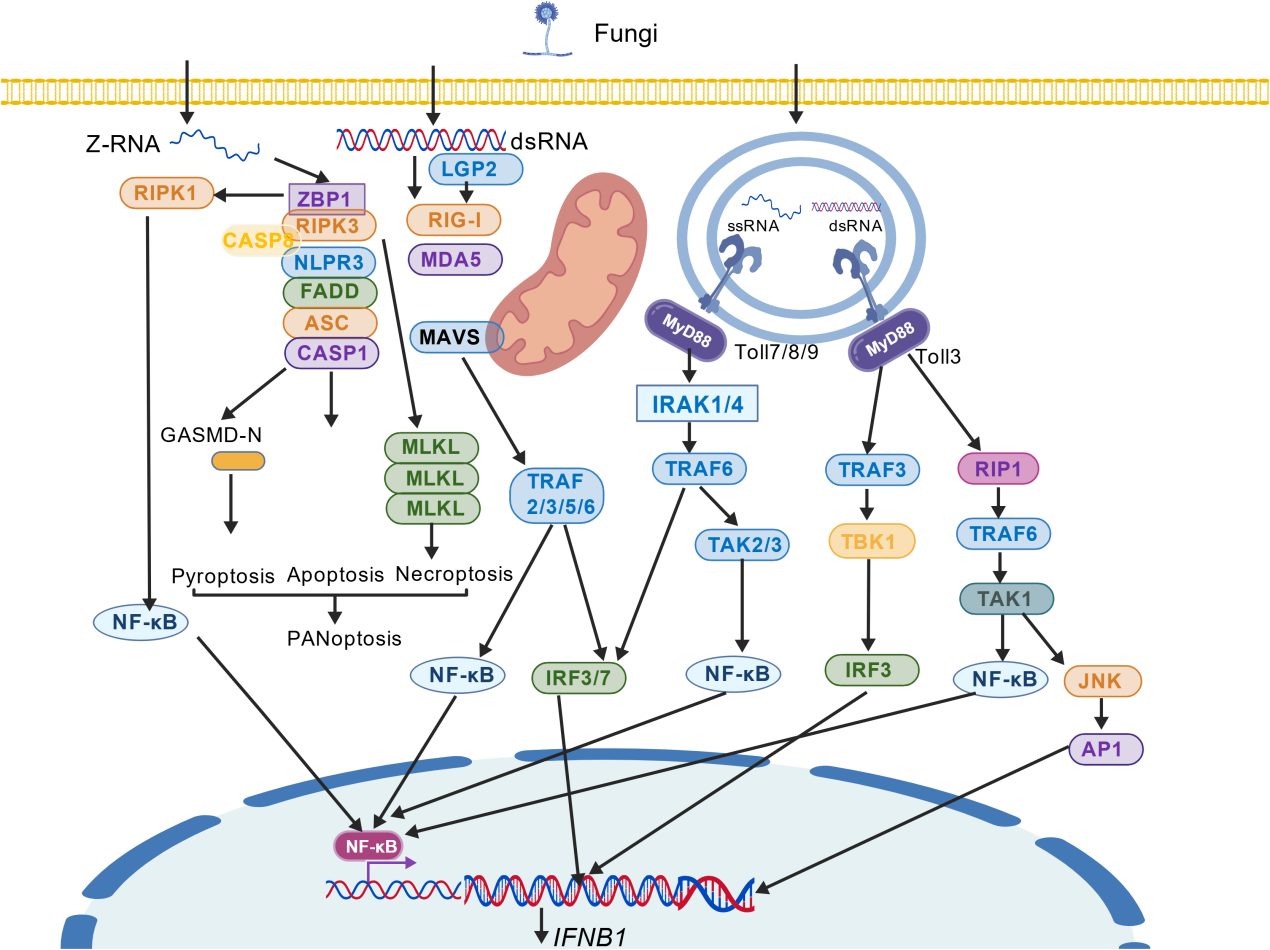
Fungal DNA sensors and their downstream signaling pathways.

The cGAS-STING pathway represents a fundamental component of the innate immune system, playing a pivotal role in the detection of aberrant DNA within the cytoplasm, thereby initiating an immune response. Cyclic GMP-AMP synthase (cGAS), a critical component of this pathway, possesses a nucleotide transferase domain and is capable of binding to the positively charged surface of either pathogens or self-dsDNA phosphate backbones. This can promote the production of the key intermediate product 2’3’-cyclic GMP-AMP (cGAMP). Stimulator of interferon genes (STING) is a transmembrane protein localized to the endoplasmic reticulum (ER), containing an N-terminal transmembrane region and a C-terminal domain (CTD) that binds cGAMP. Upon receiving cGAMP signals, the sting protein is activated to release and transport, ultimately migrating to the Golgi apparatus.There, STING recruits the kinase TBK1, promoting TBK1 autophosphorylation (18, 19) and recruiting IRF3. TBK1 phosphorylates IRF3, forming dimers that enter the nucleus to initiate type I interferon gene expression (20, 21).Concurrently, it activates the NF-κB pathway, inducing the production of proinflammatory factors such as IL-6 and TNF-α (**Figure 1**). Furthermore, STING activation has been shown to induce the recruitment of LC3 autophagy-related proteins, which play a crucial role in the clearance of intracellular pathogens (22).

A mounting body of evidence points to a close association between cGAS-STING signaling and fungal infections, with *Candida albicans* and *Aspergillus fumigatus*-induced fungal infections being the most extensively studied. In their study, Mei, Peng, and their team discovered that by impeding cGAS, it is possible to enhance the expression of non-type I interferon inflammatory genes. This, in turn, has the effect of intensifying the host’s inflammatory response to *Aspergillus fumigatus*-induced invasive pulmonary aspergillosis. This, in turn, can result in increased susceptibility to infection and impaired fungal clearance (6). Furthermore, the cGAS-STING signaling pathway has been implicated in the inflammatory response of *Aspergillus fumigatus* keratitis. *Aspergillus fumigatus* activates this pathway to promote the secretion of inflammatory factors by corneal cells, thereby exacerbating the inflammatory response. Concurrently, cGAS also plays a pivotal role in the autophagy of corneal cells induced by *Aspergillus fumigatus (*23). Contrary to the aforementioned role, research indicates that in fungal infections like *Candida albicans*-induced keratitis, STING inhibition exacerbates inflammatory progression. This phenomenon may be attributed to the observation that the inhibition of cGAS/STING signaling has been demonstrated to promote NLRP3 inflammasome activation and pyroptosis, consequently exacerbating keratitis (24). Investigating this phenomenon, Chen and Tian discovered in 2023 that *Candida albicans* infection causes STING to translocate to phagosomes together with the endoplasmic reticulum. This migration has been shown to prevent Src recruitment and Syk phosphorylation, thereby indicating that STING plays a role in the host’s defense against pathogens (25). Recent studies have demonstrated that *Candida albicans* activates the STING pathway in macrophages by delivering fungal DNA to the cytoplasmic solute of macrophages via EVs, thereby triggering the involvement of cGAS and the initiation of downstream signaling. This enhanced understanding of the type I interferon response induced by *Candida* contributes to our current knowledge in this field (5). The cGAS-STING pathway is vital for immunity against fungi. Its outcome depends on the type of fungus and the body part affected. So, to treat patients effectively, future medicines based on this pathway must take these differences into account.

#### 2.1.2 AIM2

Absent in melanoma 2 (AIM2) is a cytosolic DNA sensor that initiates inflammasome assembly. Its HIN200 domain at the C-terminus binds directly to double-stranded DNA (dsDNA), which relieves autoinhibition and promotes AIM2 oligomerization (26). The N-terminal PYD domain then recruits the adaptor protein ASC via homotypic PYD-PYD interactions. The assembled AIM2-ASC inflammasome serves as a platform for recruiting and activating caspase-1 through CARD-CARD interactions (7, 27). Active caspase-1 cleaves the precursors of IL-1β and IL-18 to mature these cytokines (28, 29), and also cleaves gasdermin D (GSDMD), whose N-terminal fragments form pores in the plasma membrane, leading to pyroptosis and the release of inflammatory mediators (30–32). (**Figure 1**).

Research on AIM2 in fungal infections highlights its collaborative role with NLRP3 in inducing the assembly of inflammasomes during *Aspergillus fumigatus* infection (9). The resulting complexes activate caspase-1 and recruit caspase-8 to promote the maturation of IL-1β and IL-18. This pathway is crucial for host defense, as evidenced by the more severe pulmonary lesions and higher fungal loads observed in AIM2-deficient mice.These mice also have significantly reduced levels of inflammatory cytokines and immune cell recruitment. These findings suggest that the AIM2 inflammasome plays an important protective role in anti-*Aspergillus immunity*. Additionally, both humans and mice exhibit liver dysfunction following infection with *Aspergillus niger*. Studies have shown that *Aspergillus niger* may activate the AIM2-caspase-1/-4-GSDMD axis in hepatocytes of mice, resulting in hepatocyte apoptosis (33). Recent studies have found that AIM2 promotes macrophage apoptosis through the AKT signaling pathway, thereby enhancing *Candida albicans* infection (8). The present study demonstrates that *Candida albicans* infection can induce the expression of the AIM2 gene in innate immune cells or tissues of humans and mice. AIM2 has been demonstrated to exert a negative regulatory effect on AKT activation and enhance macrophage apoptosis, thereby influencing the host’s defense against *Candida albicans*. Inflammasomes, including AIM2, have been demonstrated to play a role in the immune cell activity during *Candida* infection and host survival in systemic fungal infections. In summary, the AIM2 inflammasome has a double effect in fungal infections, causing either protection or disease. It is vital to understand how this happens and why, especially the switch from protection to disease. This is important to determine if AIM2 can be used to treat patients.

#### 2.1.3 TLR9

Toll-like receptor 9(TLR9) is a pivotal Nucleic acid sensor that is situated on the endosomal membrane, with a primary function of recognizing unmethylated CpG motifs in pathogen DNA, a distinct DNA sequence pattern (34). The extracellular domain of TLR9 contains leucine repeat sequences that are responsible for DNA binding, while the intracellular domain contains a conserved TIR domain for signal transduction (35). When pathogens such as fungi or bacteria are phagocytosed, their DNA is released within the endosome and recognized by TLR9 (36, 37). The recognition process is contingent upon the acidic environment of the endosome, which induces a conformational change in TLR9 and recruits the adaptor protein MyD88 (38). MyD88 has been shown to recruit the IRAK kinase family via its death domain, which in turn activates TRAF6. This process ultimately drives the activation of the NF-κB transcription factor and promotes the production of pro-inflammatory factors. Furthermore, it has been demonstrated that this process can activate the IRF7 transcription factor, which, in turn, induces the secretion of type I interferon (39, 40). Collectively, these signals orchestrate the activation of innate immune cells and inflammatory responses (**Figure 1**).

TLR9 exerts a dual role in the context of antifungal immunity. Research has demonstrated that TLR9 plays a pivotal role in the host’s recognition of fungi, such as *Candida albicans*. The presence of *Candida* DNA has been shown to be detected by TLR9 in the endosomes of macrophages and dendritic cells. This detection activates downstream signals, which in turn enhance the secretion of inflammatory factors and phagocytic function. This, in turn, serves to limit the spread of the fungus (41, 42). It is noteworthy that TLR9 frequently operates in conjunction with TLR7. A study of *Histoplasma capsulatum* infection revealed that double knockout mice lacking both TLR7 and TLR9 (TLR7/9^−/&−^) exhibited a complete abrogation of the protective IFN-I response, severe fungal dissemination to the brain, and significantly higher mortality compared to wild-type mice. In contrast, single TLR7 or TLR9 deficiency had a less pronounced effect, highlighting a synergistic role for these receptors in host defense (43).For instance, in a murine model of *Candida* infection, TLR9-deficient mice demonstrated increased fungal loads and diminished survival rates. However, contradictory findings have emerged from further studies, which suggest that TLR9 may not be indispensable in systemic fungal infections and may even exhibit functional redundancy (44, 45). Its protective effects are hypothesized to vary depending on the fungal species, the infection site, and the host cell type. The identity of the host cell is of particular importance. Van Prooyen et al. identified a specific DC subset, CD103+ conventional dendritic cells (cDCs), as the major producer of IFN-I in the lungs of mice infected with *Histoplasma capsulatum*. This finding ascribes a previously underappreciated role to CD103+ cDCs in antifungal immunity and provides a cellular basis for the TLR7/9-dependent protective response (43). These disparities may be associated with the methylation patterns of fungal DNA or the maturation status of host endosomes. Notably, artificially synthesized TLR9 agonists have demonstrated potential in preclinical studies by enhancing immune cell activation and Th1-type immune responses, thereby improving host antifungal capacity. Consequently, the targeting of the TLR9 pathway offers potential for the development of novel immunomodulators or combination therapies (38, 45).

Upon entry into the cell, fungal DNA is sensed by the cytosolic nucleic acid sensor cGAS. cGAS activation initiates downstream signaling that induces the expression of type I interferon genes, while concurrently triggering the NF-κB signaling pathway to launch antifungal effector responses. Additionally, the AIM2 protein can recognize fungal DNA, leading to the activation of caspase-1. This process results in the maturation and secretion of the pro-inflammatory cytokines IL-1β and IL-18, and cleaves GSDMD-N to promote pyroptosis.TLRs that recognize fungal nucleic acids activate the TRAF6 protein, leading to the concurrent induction of type I interferon gene expression and initiation of the NF-κB signaling pathway.

### 2.2 RNA sensors

#### 2.2.1 ZBP1

**Figure 2:**
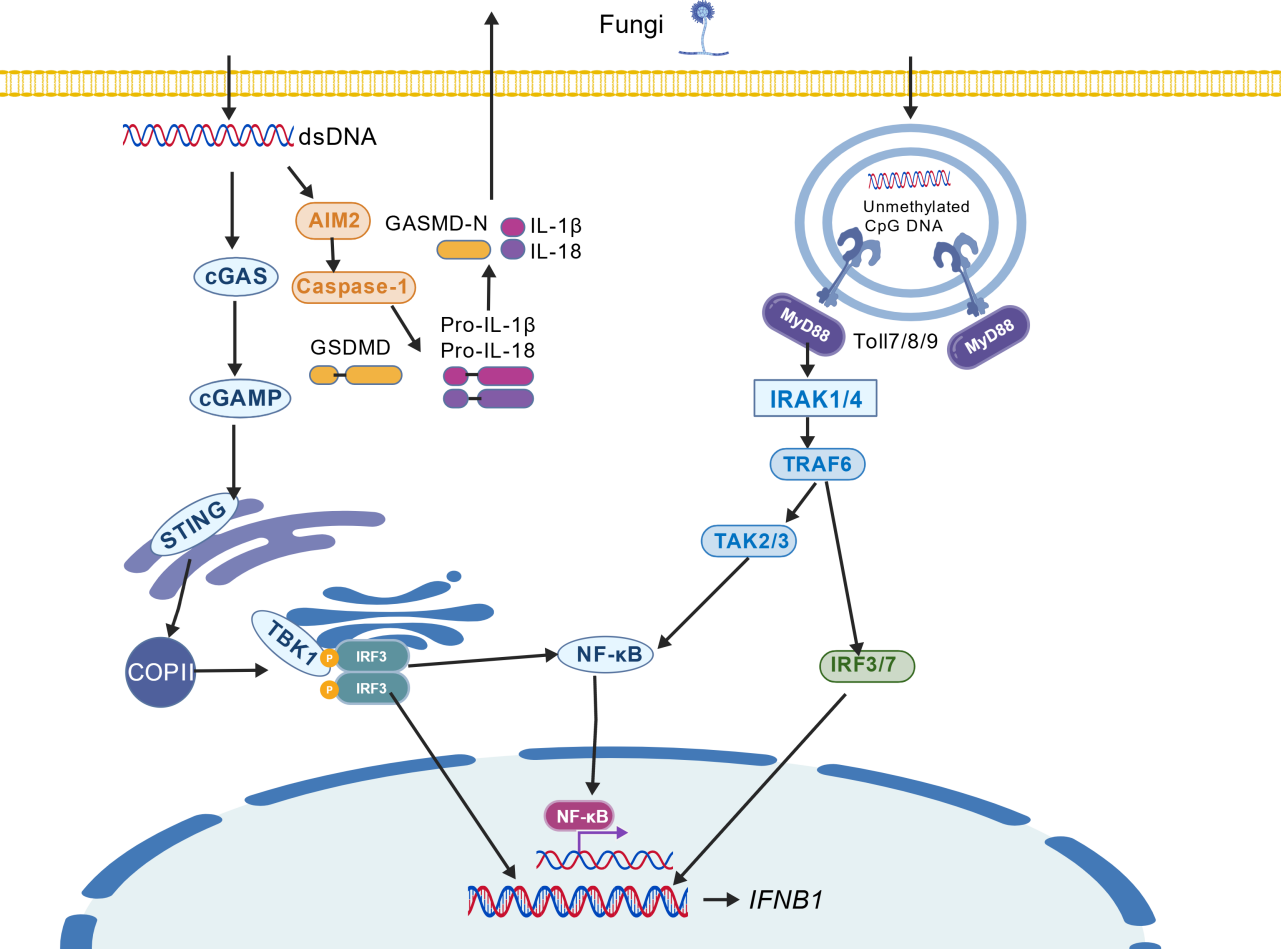
Fungal RNA sensors and their downstream signaling pathways.

Z-DNA Binding Protein 1 (ZBP1) is a cytosolic nucleic acid sensor pivotal for innate immunity. Although it recognizes both Z-DNA and Z-RNA, it is classified as an RNA sensor based on its primary role in detecting Z-RNA during fungal infections. Its N-terminal Zα and Zβ domains specifically bind left-handed Z-form nucleic acids (33, 34). Upon ligand engagement, ZBP1 initiates dual signaling pathways via its C-terminal region: it recruits TBK1 to phosphorylate IRF3, driving IRF3 dimerization, nuclear translocation, and type I interferon production; concurrently, it employs its RHIM domains to interact with RIPK1 and RIPK3, thereby activating the NF-κB pathway and facilitating the assembly of the PANoptosome—a multiprotein complex that coordinates programmed cell death and inflammatory signaling (36, 46, 47). Notably, ZBP1-mediated Z-RNA sensing can also occur within the nucleus during certain viral infections (33). (**Figure 2**).

ZBP1 plays a pivotal role in the immune response to various infections, including those of a fungal nature. *Candida albicans* and *Aspergillus fumigatus* have been observed to induce PANoptosis in cells, and the Zα2 domain of ZBP1 has been identified as being essential for promoting inflammasome activation and PANoptosis. This suggests the potential for ZBP1 to play a role in the host’s defense against fungal pathogens (10). Furthermore, in the context of oral mucosal diseases induced by *Candida albicans* infection, ZBP1 has been shown to elicit the ZBP1-RIPK3/NLRP3/caspase-8-mediated apoptosis pathway, thereby impeding the progression of the disease (38). Notably, ZBP1 has been characterized as a multifaceted molecule in immune responses, capable of both promoting host defense against pathogens and contributing to inflammatory pathologic progression (39). Subsequent to influenza A virus (IAV) infection, ZBP1 instigates RIPK3-mediated MLKL activation within the cell nucleus, consequently stimulating neutrophils and exacerbating pneumonia in murine models (40). In essence, ZBP1 operates as an upstream sentinel in the context of fungal infections, thereby translating the detection of Z-nucleic acid into the orchestrated programmed cell death known as PANoptosis. This distinct role differentiates it from other cytosolic sensors and underscores the activation of complex cell death pathways as a critical, albeit not yet fully comprehended, component of antifungal immunity.

#### 2.2.2 MDA5

Melanoma Differentiation-Associated protein 5(MDA5) is composed of two CARD domains at the N-terminus, an intermediate helicase domain, and a C-terminal regulatory or inhibitory domain. In its initial state, the CARD domains of MDA5 adopt an open conformation, enabling them to bind to long-chain K63-polyUb (48). MDA5 predominantly recognizes long-chain dsRNA, with a size that is typically longer than 1000 base pairs. The C-terminal regulatory domain has been observed to form unstable filaments with long-chain dsRNA, and the RNA helicase domain has been shown to play a crucial role in recognizing long-chain dsRNA (49, 50). Following the recognition of dsRNA, MDA5 binds to and hydrolyzes ATP. Its RNA-dependent ATPase activity has been shown to allosterically regulate the stability of the MDA5-CARDs complex, which is mediated by K63-polyUb (48). Following the recognition of dsRNA, MDA5 binds to and hydrolyzes ATP. Its RNA-dependent ATPase activity has been shown to allosterically regulate the stability of the MDA5-CARDs complex, which is mediated by K63-polyUb (51).(**Figure 2**).

MDA5 plays a critical role in innate immunity, including responses to fungal pathogens. Davis et al. demonstrated that MDA5 signaling increases pulmonary vascular permeability, which facilitates the leakage of antimicrobial serum factors and likely promotes immune cell infiltration, thereby protecting mice from opportunistic fungal infections (52). Conversely, Chen et al. revealed a pathogen-enhancing mechanism, showing that MDA5 activation promotes macrophage apoptosis via regulation of Noxa, Bcl2, and Bax. Critically, this MDA5-driven apoptosis—coupled with iNOS suppression that blunts macrophage killing—directly supports a key pathogenic strategy of *C. albicans*, which actively induces host cell death to promote its own dissemination (11). Clinically, anti-MDA5 dermatomyositis is a distinct subtype of inflammatory myopathy characterized by the presence of autoantibodies against MDA5 and is frequently associated with a high risk of rapidly progressive interstitial lung disease. Within this patient population, CD8+ T-cell exhaustion is significantly associated with a heightened risk of pulmonary fungal infection (53). Furthermore, fungal infection is an independent risk factor for spontaneous mediastinal emphysema (54) and predicts increased mortality in MDA5-associated interstitial lung disease (55). MDA5 has dual identity as a pathogen sensor and autoantigen. Anti-MDA5 dermatomyositis patients show severe fungal susceptibility. The greatest clinical significance of MDA5 may lie in defining risk and guiding prophylaxis in this specific population. Future research must differentiate between its functions in general host defence and its role in infection risk in autoimmune contexts.

#### 2.2.3 TLR7/8

Toll-like receptor 7(TLR7) and Toll-like receptor 8(TLR8) are pattern recognition receptors localized to the endosomal membranes of immune cells, where they specifically recognize single-stranded RNA (ssRNA) from pathogens. TLR7 and TLR8 differ in ligand specificity, cellular expression and function. TLR7 responds to guanosine/uridine-rich motifs, while TLR8 is activated by uridine-rich sequences. TLR7 is expressed in plasmacytoid dendritic cells and B cells, and TLR8 in myeloid cells. TLR7 signaling induces type I interferons in pDCs, whereas TLR8 activation drives pro-inflammatory cytokine production in myeloid cells.Their activation, similar to TLR9, is triggered upon phagocytosis of pathogens and the release of their genomic RNA within endosomes (56). As described for TLR9 above, ligand binding induces a conformational change that recruits the adaptor protein MyD88, initiating the canonical MyD88-dependent signaling cascade. This leads to the activation of NF-κB and IRF7 transcription factors, driving the production of pro-inflammatory cytokines and type I interferons, respectively (57, 58) (59, 60). (**Figure 2**).

In the context of the antifungal immune response, the TLR7/8 pathway fulfills a pivotal yet intricate function. Research has demonstrated that the RNA components of certain fungal pathogens can be recognized by TLR7/8 in the endosomes of host immune cells (61). This recognition activates the aforementioned signaling pathway, promoting the secretion of pro-inflammatory cytokines and type I interferons. It is important to note that the functional outcome of this recognition is highly cell-type specific. A seminal study by Van Prooyen et al. demonstrated that while dendritic cells mount a robust toll-like receptor 7/9 -dependent type I interferon response and efficiently restrict *Histoplasma capsulatum* growth, macrophages are largely incapable of such a response and are permissive for fungal replication (43). These factors are crucial for activating the phagocytic and cytotoxic functions of innate immune cells, as well as linking and initiating the adaptive immune response, thereby aiding the host in clearing fungal infections (60). The study further clarified that the TLR7/9-driven IFN-I response in dendritic cells (DCs) is critical not only for cell-intrinsic fungal restriction, but also for the subsequent activation of CD4+ T cells and the production of IFN-γ, a key cytokine for systemic host defense (43). However, the activation of TLR7/8 signaling is not invariably protective. Excessive or prolonged TLR7/8 signaling activation has been demonstrated to result in excessive inflammatory responses and tissue damage. In some cases, this activation may impair host defense or promote disease progression. Given its ability to effectively enhance antifungal immune responses, synthetically derived TLR7/8-specific agonists have been extensively studied as potential immunotherapy adjuvants or drugs, such as R848 and its derivatives (62). In preclinical models, the local or systemic application of such agonists has been demonstrated to enhance the host’s resistance to fungi such as *Candida albicans*, suggesting a potential for novel antifungal immunotherapy (63, 64). Consequently, the TLR7/8 pathway functions as a pivotal conduit between fungal recognition and host immune activation. A more profound understanding of its mechanisms, especially its cell-type-specific functions and role in orchestrating adaptive immunity, could lead to the development of new antifungal strategies (43).

#### 2.2.4 TLR3

Toll-like receptor 3(TLR3) is a pivotal nucleic acid sensor situated on the endosomal membrane, occupying a central role in antifungal innate immunity. It is a type I transmembrane protein whose structure comprises an extracellular region rich in leucine repeat sequences, transmembrane domains, and an intracellular TIR domain (65). TLR3 specifically recognizes double-stranded RNA (dsRNA), an intermediate product of replication for many RNA viruses (66). However, it is noteworthy that certain fungi have been observed to produce dsRNA during their life cycle or through viral replication, suggesting their potential to act as ligands for TLR3. Recent studies have further indicated that under homeostatic conditions, endogenous long non-coding RNA (Rmrp) can bind to TLR3 within endosomes (67). This self-RNA-mediated pre-activated state, while not directly inducing robust inflammatory responses, significantly heightens TLR3’s sensitivity to subsequent pathogen dsRNA stimuli, priming innate immune recognition. The process of TLR3 signaling is entirely dependent on the MyD88-independent pathway, also referred to as the TRIF-dependent pathway. Subsequent to ligand recognition, the intracellular TIR domain of TLR3 recruits the key adaptor protein TRIF. This process functions as a signaling platform, subsequently recruiting downstream molecules such as TRAF3, RIP1, and TBK1. This pathway ultimately activates two transcription factors, NF-κB and interferon regulatory factor 3 (68). Phosphorylation and nuclear translocation of IRF3 are critical for inducing type I interferon production, while NF-κB activation drives pro-inflammatory cytokine expression. The complete TLR3 signaling pathway is imperative for optimal host defense against fungal infections (69–71). For instance, in an *Aspergillus fumigatus* infection model, TLR3, TRIF, and IFN-β mRNA expression in infected mouse lung tissue was significantly increased, reaching its peak at 24 hours, thereby confirming effective pathway activation during infection (72, 73).(**Figure 2**).

Activation of TLR3 plays a multifaceted role in coordinating antifungal immune responses, with its core function being the bridging of innate and adaptive immunity. The process of antigen-presenting cells recognizing fungal-derived dsRNA via TLR3 has been shown to promote their maturation, upregulate the expression of major histocompatibility complex and co-stimulatory molecules, and secrete specific cytokines. This process effectively presents pathogen signals to T cells, inducing a Th1-type immune response. The Th1 response, typified by IFN-γ production, is pivotal for activating effector cells, such as macrophages, to eradicate intracellular fungi. Furthermore, stimulation of macrophages and neutrophils with the TLR3-specific agonist poly(I:C) significantly enhances their phagocytic and killing capabilities against common fungal pathogens like *Candida albicans*, providing experimental evidence for TLR3’s direct empowerment of innate immune cells (72–74). A substantial body of research, both *in vivo* and *in vitro*, has provided unequivocal evidence for TLR3’s pivotal role in antifungal defense. In a study of a respiratory infection model caused by *Aspergillus fumigatus*, researchers observed significant activation of the TLR3 signaling pathway in mouse lung tissue. The innate immune response mediated by this pathway exerted a protective effect by clearing pathogens and mitigating lung tissue damage. This protective effect may be associated with the type I interferons produced following TLR3 activation. These interferons not only directly inhibit fungal growth but also further promote fungal clearance by enhancing the activity of natural killer cells and cytotoxic T cells (75). However, the immunoprotective role of TLR3 is not without complexity, exhibiting a dual effect that can be both beneficial and detrimental. Within the TLR3-mediated inflammatory response, a positive feedback regulatory loop driven by type I interferons exists. TLR3 activation induces type I interferons via IRF3, which then binds to its own receptors through autocrine or paracrine mechanisms, activating the JAK/STAT signaling pathway and subsequently inducing upregulation of TLR3 expression itself (76). While this loop amplifies antiviral and antifungal signals, excessive or sustained TLR3 activation under specific conditions—such as in immunosuppressed hosts or in high-dose infections—can trigger excessive production of proinflammatory cytokines. This process, known as a “cytokine storm,” results in severe tissue damage and immunopathological changes that, paradoxically, favor fungal invasion and dissemination. The antifungal role of the TLR3-TRIF axis is unique among endosomal TLRs. Its distinct signalling pathway is critical for defence, but its inherent design carries the risk of immunopathology through an uncontrolled interferon feedback loop. The antifungal role of the TLR3-TRIF axis is unique among endosomal TLRs. Its distinct signalling pathway is critical for defence, but its inherent design carries the risk of immunopathology through an uncontrolled interferon feedback loop. Strategies must therefore focus on balancing the beneficial effects of TLR3 activation against the risk of inflammation.

Upon recognition of fungal Z-RNA, the ZBP1 protein induces programmed necroptosis (PANoptosis). Meanwhile, the MDA5 protein detects fungal double-stranded RNA (dsRNA) and activates the downstream adaptor MAVS. This, in turn, triggers the NF-κB signaling pathway and potentiates the activation of type I interferon responses.TLRs that recognize fungal nucleic acids activate the TRAF3 and TRAF6 protein, leading to the concurrent induction of type I interferon gene expression initiation of the NF-κB signaling pathway and activate JAK/STAT signaling pathway.

## 3 Regulation of nucleic acid sensors and immune evasion

### 3.1 Host regulatory mechanisms

In the context of antifungal immunity, host cells meticulously regulate the activity of nucleic acid sensors through a multitude of mechanisms. This regulatory process is pivotal in maintaining a balance between the effective clearance of pathogens and the prevention of excessive immune responses. Fungal nucleic acids, as pathogen-associated molecular patterns, are recognized by specific nucleic acid sensors in the host cytoplasm or endosomes, thereby triggering downstream signaling pathways. Hosts regulate the intensity and duration of immune responses through a variety of mechanisms, including ubiquitin modification, autophagy pathways, subcellular compartmentalization regulation, and feedback mechanisms. The complexity of this regulatory network is comparable to that of viral nucleic acid recognition systems. It is noteworthy that host regulatory mechanisms manifest not only at the molecular level but also exhibit significant cell type specificity. For instance, despite their shared designation as professional phagocytes, dendritic cells and macrophages exhibit notable disparities in the intensity of nucleic acid sensor pathway activation and the ensuing functional outcomes. This further underscores the intricate and nuanced regulatory dynamics that characterize the host’s immune response (43).

#### 3.1.1 Ubiquitination modification and autophagy regulation

The process of ubiquitination of proteins plays a pivotal role in the regulation of the stability and activity of nucleic acid sensors. Hosts achieve targeted degradation or signal activation by ubiquitinating these sensors via E3 ubiquitin ligases. For instance, the E3 ubiquitin ligase TRIM11 undergoes auto-ubiquitination at the K458 site, delivering the DNA sensor AIM2 to autophagosomes for degradation, thereby suppressing excessive inflammasome activation (77). Concurrently, K63-linked polyubiquitination, mediated by TRAF6, has been shown to positively regulate TLR signaling, thereby enhancing antifungal immunity (78, 79). This bidirectional regulatory mechanism enables the host to precisely modulate immune response intensity according to the infection stage. The autophagy pathway plays a crucial role in regulating the intensity of nucleic acid sensor signaling. The cGAS-STING signaling pathway is subject to a self-limiting degradation process that serves to regulate its function. STING activation has been shown to induce the recruitment of the autophagy-associated protein LC3 (80), ultimately leading to STING degradation via autophagosome-lysosome fusion, thereby limiting signaling duration. This autophagy-mediated negative feedback represents a crucial mechanism that prevents sustained immune responses from causing autoimmune damage. Recent studies have also revealed close links between metabolic pathways and NCRO signaling. Purinic metabolic enzymes, including adenosine deaminase, fulfill a pivotal negative regulatory function within the context of innate immunity. A deficiency in purine recycling enzymes results in the accumulation of the purine metabolite deoxyadenosine. This, in turn, activates immune gene expression and pathogen resistance (81). This provides a novel perspective on the metabolic regulation of nucleic acid sensors.

#### 3.1.2 Synergistic effects of subcellular compartmentalization and signaling pathways

Hosts precisely regulate the activity of nucleic acid sensors through subcellular compartmentalization strategies. TLR3, TLR7, TLR8, and TLR9 within endosomes have been shown to recognize fungal nucleic acids. However, the activation of these receptors strictly depends on endosomal maturation and the presence of an acidic environment. The efficiency with which sensors interact with ligands and the intensity of downstream signaling are influenced by the hosts, who regulate endosomal transport, fusion, and acidification processes with precision. This compartmentalized recognition mechanism is a critical strategy employed by hosts to differentiate between self and foreign nucleic acids, thereby averting excessive activation of autoimmune responses. Recent studies have revealed that TLR7/9-mediated nucleic acid recognition plays a pivotal role in antifungal immunity within specific dendritic cell (DC) subsets, such as CD103+ cDCs. During infection with *Histoplasma capsulatum*, CD103+ cDCs—rather than macrophages or plasmacytoid DCs—have been identified as the primary cellular source of the type I interferon response in lung tissue. This response is contingent upon the presence of TLR7/9 and its downstream adaptor protein MyD88. TLR7/9 deficiency has been shown to result in impaired IFN-I production, reduced fungal clearance capacity, and significantly increased host mortality. This finding indicates that the function of endosomal nucleic acid sensors exhibits critical cell type specificity and suggests that CD103+ cDCs represent a previously underappreciated key pathway in immunity against pulmonary fungal infections (43). There is considerable evidence to suggest that there are significant synergistic interactions between different nucleic acid sensor pathways. In the context of fungal infections, it has been observed that multiple receptors can be simultaneously activated, thereby giving rise to intricate signaling networks. For instance, cGAMP, produced by cGAS upon recognition of fungal DNA, activates the STING pathway, inducing type I interferon production. Concurrently, the AIM2 inflammasome is also activated, promoting the maturation and secretion of IL-1β and IL-18. This synergistic response ensures the efficacy and diversity of the immune response, maximally suppressing fungal growth and spread. It is important to note that nucleic acid sensor signaling pathways also engage in extensive cross-talk with other immune signaling pathways. For instance, in Dectin-1-mediated antifungal signaling, multiple interactions exist between Syk-dependent signaling pathways and nucleic acid sensor pathways. TLR7/9-mediated IFN-I production is equally crucial for bridging innate and adaptive immunity. Research has demonstrated that TLR7/9-deficient dendritic cells (DCs) exhibit impaired capacity to effectively activate CD4+ T cells and induce interferon-γ (IFN-γ) production *in vitro*. In murine models of infection, TLR7/9 deficiency has been observed to result in a decline in IFN-γ and IL-12 p70 levels, accompanied by a reduction in CD69 expression, a marker of T cell activation (43). This cross-regulatory mechanism is indicative of the intricate design of the host immune system, ensuring that an appropriate and effective immune response is generated against various types of fungal infections.

#### 3.1.3 Regulation of programmed cell death

Programmed cell death is a critical component of host defense mechanisms and plays a pivotal role in antifungal immunity. It has been demonstrated that nucleic acid sensors induce cytokine production and activate multiple programmed cell death pathways, including pyroptosis, apoptosis, and necrotic apoptosis. During the course of a fungal infection, the activation of inflammasomes such as AIM2 has been observed to promote caspase-1-mediated pyroptosis, resulting in the demise of infected cells and the direct elimination of sites of fungal replication (82). Recent studies have revealed complex cross-regulatory interactions among different forms of programmed cell death, forming intricate cell death networks. The induction of ZBP1 expression is initiated by type I interferons. The subsequent activation of ZBP1-mediated necrosis and inflammation serves to enhance the production of type I interferons, thereby establishing a positive feedback loop that amplifies the host antifungal immune response (83). Conversely, the role of caspase-8 is to negatively regulate ZBP1-mediated RIPK3 activation by degrading components of the ZBP1–RIPK3 complex, thereby preventing excessive tissue damage (84). This delicate balance is critical to ensure the efficacy and safety of immune responses.

### 3.2 Fungal strategies for immune evasion

Fungal pathogens have evolved a sophisticated array of immune evasion mechanisms over long-term evolutionary processes to circumvent immune recognition mediated by host nucleic acid sensors. These strategies encompass the construction of physical barriers, active disruption of immune signaling, targeted escape from specific nucleic acid sensors, and population-level adaptation. These strategies reflect the complex coevolutionary relationship between fungi and host immune systems.

#### 3.2.1 Cell wall modification and physical barriers

Fungi evade recognition by the immune system of their hosts by dynamically modifying their cell wall structure and composition, thereby reducing exposure of molecular patterns associated with pathogens. *Candida albicans* has been shown to impede recognition by host cell surface receptors, such as Dectin-1, through the increased production of mannan in the outer cell wall layer. This, in turn, serves to shield the inner β-glucan layer, thereby creating a barrier to recognition (85).The inherent melanin synthesis in *Cryptococcus neoformans* serves to establish a physical barrier that hinders not only DNA release but also phagosome acidification, thereby impeding TLR9 activation within endosomes (86). These modifications effectively weaken nucleic acid sensor activation by reducing fungal nucleic acid exposure.

#### 3.2.2 Active immune interference and molecular mimicry

In addition to passive physical barriers, fungi secrete effector molecules that disrupt host immune signaling. *Candida albicans*, a fungal pathogen, has been observed to secrete aspartic proteases that have the capacity to degrade various host antibodies, complement components, and proinflammatory cytokines. *Aspergillus fumigatus*, a fungus, produces a substance called melittin, which has been shown to inhibit NF-κB activity and block downstream proinflammatory signaling pathways. Recent studies have revealed that *Candida albicans* effector Sce1 binds to cell wall β-glucans via its PIR motif, thereby masking its immunogenicity. Following phagocytosis by immune cells, it induces caspase-dependent apoptosis, executing a dual escape strategy of “evasion followed by attack” (87). With regard to the concept of molecular mimicry, various fungal species employ divergent strategies. Extracellular vesicles (EVs) derived from *Candida albicans* have been observed to infiltrate the membranes of host cells, thereby impeding the signaling process of type I interferons (IFNs) by modulating the cGAS-STING pathway (47). In contrast, *Cryptococcus neoformans* vesicles exhibit a distinct outer structure that hinders macrophage internalization, thereby diminishing immune activation (88). This species-specific regulation exemplifies the precise adaptability of fungal escape strategies.

#### 3.2.3 Specific escape from targeted nucleic acid sensors

Fungi have evolved sophisticated mechanisms to directly interfere with nucleic acid sensor signaling pathways. Research has demonstrated that during *Candida albicans* infection, the STING protein undergoes an aberrant transport process, deviating from the typical trajectory of the Golgi apparatus and instead arriving at the phagosomes. STING exerts its function by binding to Src kinase via its N-terminus, thereby inhibiting Syk phosphorylation and consequently negatively regulating Dectin-1-mediated antifungal immunity (25). Of particular interest is the novel mechanism of B-cell linker protein (BLNK) in immune evasion. Researchers at Tongji University have determined that *Candida albicans* activates BLNK in macrophages via cell wall polysaccharides, thereby competitively inhibiting c-Cbl phosphorylation. This results in the inhibition of cytoskeletal assembly and pseudopod formation, leading to a substantial suppression of macrophage migration to sites of infection. During the course of a fungal infection, the absence of BLNK in mice resulted in heightened pathogen clearance and augmented survival rates (89). This finding unveils a novel fungal strategy to evade nucleic acid sensor recognition by disrupting immune cell migration.

#### 3.2.4 Biofilm formation and collective defense

At the population level, fungi enhance their immune evasion capabilities by forming biofilms. The three-dimensional structure of biofilms provides robust physical protection for the fungal cells within, acting as a physical barrier that effectively prevents the release of fungal nucleic acids and avoids detection at the source. Fungal extracellular vesicles have been demonstrated to exert a multitude of effects on biofilms. Research indicates that specific cargo proteins within vesicles are crucial for assembling biofilm matrix and facilitating fungal adhesion and spreading in *Candida albicans* biofilms (90). Furthermore, fungi exhibit remarkable environmental adaptability at the population level. Research has demonstrated that *Candida albicans* meticulously calibrates environmental pH alkalization through the synergistic operation of transcription factors Dal81 and Stp2, a process that is indispensable for intestinal colonization and systemic infection (91, 92). The dual-gene knockout strain exhibits a complete loss of colonization ability and virulence, suggesting the pivotal function of this regulatory network in fungal host adaptation. This capacity for adaptability at the population level facilitates the coordination of behaviors among the fungus, thereby enhancing its overall resistance to drugs and its ability to evade immune systems.

## 4 Conclusion and future prospect

### 4.1 Summary of the preceding text

This review methodically elucidates the intricate network of nucleic acid sensors that form a critical defense against fungal infections by detecting fungal nucleic acids and triggering specific immune responses. A critical finding is that the function of these nucleic acid sensors is profoundly influenced by their pathophysiological environment. The type of fungus, the affected tissue, and the type of host cell involved collectively and finely regulate the direction of the immune response, ultimately determining whether it exerts a protective or pathogenic effect. Furthermore, the host employs complex post-translational modifications and cellular pathways to finely tune the intensity and duration of these responses, ensuring a balance between effective pathogen clearance and prevention of immunopathological damage. However, fungal pathogens have also evolved intricate evasion mechanisms, such as modifying cell walls to conceal pathogen-associated molecular patterns or hijacking host regulatory nodes. This creates a dynamic interplay between host defense and pathogen escape. Current research endeavors are focused on elucidating the precise functions of these pathways within specific tissues and cell types. Additionally, research is being conducted to decipher the double-edged sword effect of their overactivation, which can lead to immunopathology. It is imperative to note that conventional antifungal medications target a singular pathway. Their extensive utilization has precipitated the emergence of profound drug resistance concerns, thereby impeding the efficacious management of clinical treatments. In summary, precisely modulating the host’s innate immune recognition mechanisms—rather than directly targeting fungi—promises to be a novel paradigm for overcoming resistance. This “host-directed therapy” is predicated on the enhancement of the immune system’s capacity to identify and eradicate fungi, thus compensating for the limitations of conventional pharmaceutical agents. On the one hand, the employment of agonists targeting key pathways such as cGAS-STING has the potential to directly “activate” the innate immune system, particularly in immunocompromised patients, thereby inducing robust type I interferon responses. Nevertheless, precise regulation is imperative to avert tissue damage from excessive inflammation. Conversely, a promising strategy entails the development of novel pharmaceutical agents that disrupt fungal physical barriers, thereby indirectly enhancing immune recognition. For instance, Yu Hongjun’s team identified a fungal-specific GPI transamidase as a novel target. Interfering with this enzyme’s function disrupts cell wall integrity, thereby exposing the fungal nucleic acids concealed within (93). In a similar vein, Wang Zongqiang’s team ascertained that Mandimycin targets phospholipids within the cell membrane, thereby compromising its structural integrity. This mechanism may also promote the release of fungal nucleic acids for detection by host sensors (94). These strategies are designed to “unmask” the fungi, thereby re-exposing them to the surveillance of the host immune system. By synergizing with traditional drugs, they offer innovative approaches to combat infections caused by drug-resistant strains. Future research should prioritize the validation of the efficacy and safety of these strategies in more complex physiological models, while concurrently exploring the interactions among different nucleic acid sensor pathways. A comprehensive understanding of the molecular networks that govern host-fungal interactions is imperative for the development of immune intervention strategies that target key regulatory nodes, such as ubiquitination and autophagy. These strategies will pave new pathways to address the formidable challenge posed by clinically resistant fungal infections.

### 4.2 Outlook

In summary, cytoplasmic nucleic acid sensors regulate fungal immune responses through highly dynamic molecular networks, and their functions are dependent on pathogen type, site of infection, and host status. Future research endeavors should integrate the following three factors: pathogen escape mechanisms, host genetic background, and microenvironmental factors. Only by taking this comprehensive approach will we be able to develop precise immune intervention strategies. The targeting of ubiquitinylation regulatory nodes or key molecules in the cell death pathway may yield novel therapeutic approaches for drug-resistant fungal infections.
